# Challenges and opportunities of strain diversity in gut microbiome research

**DOI:** 10.3389/fmicb.2023.1117122

**Published:** 2023-02-17

**Authors:** Benjamin D. Anderson, Jordan E. Bisanz

**Affiliations:** ^1^Department of Biochemistry and Molecular Biology, Pennsylvania State University, University Park, PA, United States; ^2^The Penn State Microbiome Center, Huck Institutes of the Life Sciences, University Park, PA, United States

**Keywords:** strain diversity, gut microbiome, comparative genomics, metagenomics, species concept

## Abstract

Just because two things are related does not mean they are the same. In analyzing microbiome data, we are often limited to species-level analyses, and even with the ability to resolve strains, we lack comprehensive databases and understanding of the importance of strain-level variation outside of a limited number of model organisms. The bacterial genome is highly plastic with gene gain and loss occurring at rates comparable or higher than *de novo* mutations. As such, the conserved portion of the genome is often a fraction of the pangenome which gives rise to significant phenotypic variation, particularly in traits which are important in host microbe interactions. In this review, we discuss the mechanisms that give rise to strain variation and methods that can be used to study it. We identify that while strain diversity can act as a major barrier in interpreting and generalizing microbiome data, it can also be a powerful tool for mechanistic research. We then highlight recent examples demonstrating the importance of strain variation in colonization, virulence, and xenobiotic metabolism. Moving past taxonomy and the species concept will be crucial for future mechanistic research to understand microbiome structure and function.

## Introduction

Humans may be considered a holobiont: the sum of ourselves and our microbial inhabitants (Salvucci, 2016). The gastrointestinal tract is home to an extremely diverse set of taxa that are highly unique to each individual (Human Microbiome Project Consortium, 2012). Adding to this complexity is the staggering amount of diversity that occurs among microbes of the same species which is commonly referred to as intraspecific, intraspecies, or strain diversity (Truong et al., 2017; Van Rossum et al., 2020). While these strains sometimes form coherent subpopulations termed subspecies, there is a broad spectrum of diversity within species that have led some to question the relevance of the species concept as it pertains to prokaryotes (Fraser et al., 2009). Recent estimates indicate that the gut microbiome is home to ~4,644 bacterial species that are a reservoir for ~170 million genes (Almeida et al., 2021). Based on these estimates, a simplistic calculation would suggest the average bacterial species contains ~35,000 genes, an order of magnitude more than what is observed in most bacterial genomes (Land et al., 2015). The discrepancy between these two estimates underlies a fundamental principle of bacterial genetics and challenges the relevance of the species concept itself in gut microbes.

## Strain diversity and its origins

The species boundary in higher order sexually reproducing eukaryotes is relatively simple to qualify; however, the same cannot be said of prokaryotes. Early attempts to identify bacterial species were predominantly based on morphological observations and biochemical assays (Vandamme et al., 1996). Later, the molecular approach of DNA–DNA hybridization helped define species (De Ley et al., 1970) which was eventually supplanted by the more accessible sequencing of the 16S rRNA gene (Woese and Fox, 1977). These later methods provided objective quantitative thresholds for species: 70% hybridization and 97% identity, respectively. 16S rRNA gene sequencing has been a mainstay of taxonomists for the greater part of 30 years; however, rapid developments in whole genome sequencing enabled the comparison metric of whole genome average nucleotide identity (ANI) wherein ≥94–95% has been generally accepted as the species boundary (Konstantinidis and Tiedje, 2005). Despite the widespread adoption of these quantitative boundaries, modern taxonomy is still ripe with inconsistencies such as *Shigella* spp. being a subspecies of *Escherichia coli* and the polyphyletic nature of the genus *Clostridium* (Lan and Reeves, 2002; Yutin and Galperin, 2013; The et al., 2016).

**Figure 1:**
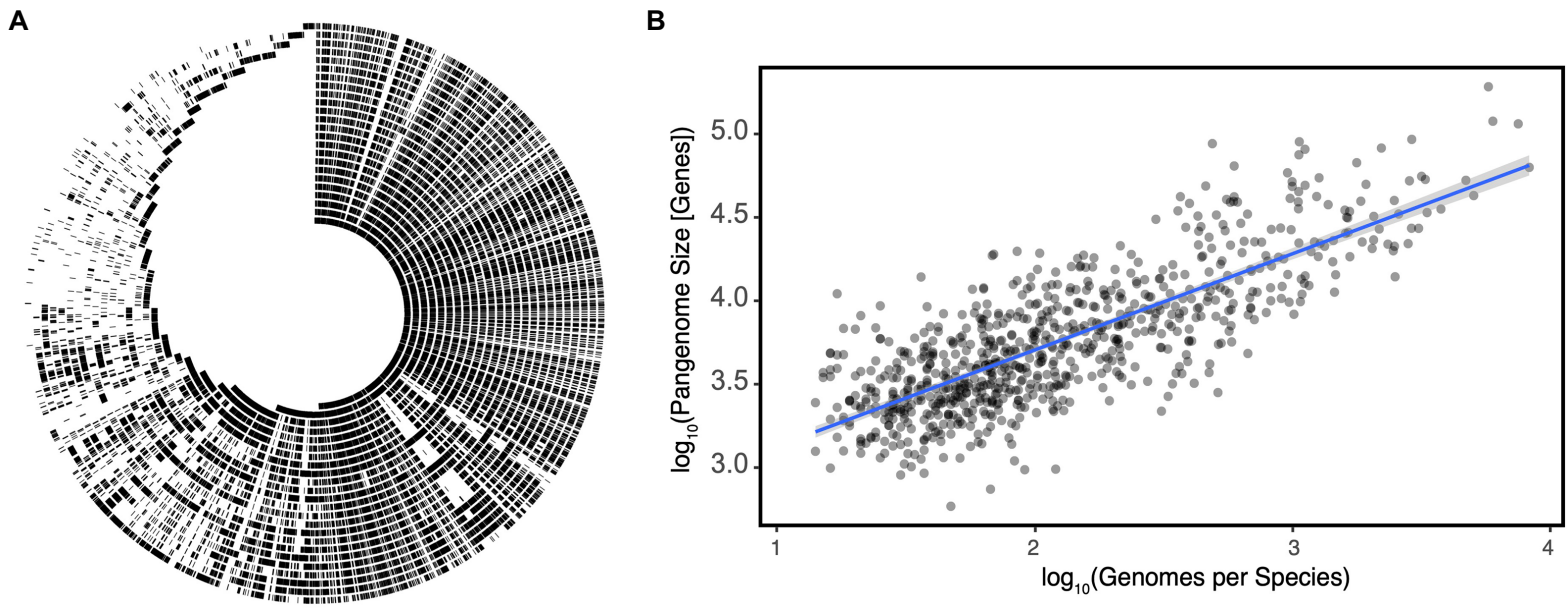
**(A)** An alignment of individual *Eggerthella lenta* strain genomes demonstrates significant variation in presence/absence of large genetic islands within the species as a function of strain. Each track represents the genome of an individual strain and with shared regions indicated in black. **(B)** Pangenome size increases linearly with the number of genomes sequenced per species in gut microbes. Each point represents a species with the blue line representing a linear regression ±SE. Data for **(A,B)** reproduced from Bisanz et al. (2020) and Almeida et al. (2021), respectively.

The ANI definition leads to a common misunderstanding: it is not to say that 95% of the genome is the same; rather, the portions of the genome which are shared have on average ≥ 95% nucleotide identity. This subtle difference has a not-so-subtle effect on how we interpret the meaning of a species as it masks the important observation that members of the same species share only a fraction of the genome with their closest relatives (Figure 1A). The set of genes found within a species is referred to as its pangenome, which can be split into the core genome: those conserved among all members of the species, and the accessory genome: those which are variably present (Medini et al., 2005). Pangenomes can vary significantly in size, but until the recent explosion of metagenome assembled genomes (MAGs), estimates were only known for a limited number of organisms with a strong bias toward model organisms and pathogens for which a ubiquity of strains had been sequenced. *Escherichia coli* for example has a core genome of ~2,000 genes, but a given strain can have an additional 1,900–3,800 accessory genes resulting in a pangenome that may be as large as 75,000 genes (Denamur et al., 2021). Alternatively in *Bacillus anthracis,* a spore-forming pathogen, the pangenome is much smaller, with a much larger core genome of ~4,000 genes, but a pangenome size of only 6,066 genes (Kim et al., 2017; McInerney et al., 2017). It is hypothesized that part of the difference in pangenome size can be attributed to differences in mutation and horizontal gene transfer (HGT) rates between species and how recently the species emerged (Segerman, 2012; Kim et al., 2017). It should however be noted that the *B. anthracis* example may illustrate another taxonomic inconsistency. *B. anthracis* may in fact be a subspecies of *B. cereus* based on ANI and a variety of other genetic approaches (Helgason et al., 2000). Indeed, re-analysis of estimates derived from MAGs indicates that a species’ pangenome grows near linearly with the number of sequenced genomes within that species (*Rho* = 0.6204, *p* = 2.2E-16, Figure 1B).

While *de novo* point mutations are common in bacterial genomes, gene gain and loss events have been estimated to occur at rates up to 4.4 fold higher (Guttman and Dykhuizen, 1994; Vos et al., 2015). These observations demonstrate that the bacterial genome is highly plastic and prone to rapid remodeling over time scales incomprehensible when thinking about genome evolution in eukaryotes. Horizontal gene transfer (HGT) is a major driver of bacterial genome evolution wherein foreign genetic material is either incorporated into the genome or maintained on mobile elements such as plasmids (Soucy et al., 2015). Horizontal gene transfer occurs through three major routes: transformation, transduction, and conjugation (Soucy et al., 2015). Transformation involves the uptake of free DNA from the environment without direct interaction between bacteria (Blokesch, 2016). Transduction is an almost accidental process by which a bacteriophage includes DNA from a donor bacterium during virion packaging, this DNA is then transferred to a recipient bacterium (Schneider, 2021). Alternatively, conjugation is a direct transfer of DNA between microbes *via* pili, which connect cells and is most commonly associated with the transfer of plasmids (Thomas and Nielsen, 2005). These traits have been well studied in the context of transferring antibiotic resistance (Lopatkin et al., 2016; Lu et al., 2017); however, they likely drive much of the genotypic variation among closely related organisms.

Just as genes can be gained through HGT, they can be lost through reductive evolution (Batut et al., 2014) or conversion to pseudogenes (Bolotin and Hershberg, 2015). Maintaining extra genetic material is energetically costly, and if they provide no benefit, they can be quickly lost. Most often gene loss is more pronounced in pathogenic strains of bacteria which have become dependent on association with their host (Moran, 2002). Essentiality describes genes that are required for an organism to live and proliferate. Screening methods have determined that only around 10% of genes in the *E. coli* genome are essential in rich media conditions (Albalat and Cañestro, 2016). This can also lead to the domesticated lab strain phenomenon wherein organisms can acquire new mutations and lose important genetic islands after repeated passaging on rich media (Sybesma et al., 2013; Denamur et al., 2021; Monteford et al., 2021). Additionally, as strains are separated by time and space, some amount of genetic drift can occur further differentiating strains (Bolotin and Hershberg, 2016). Recent experimental models investigated *E. coli* evolution in the context of host colonization following antibiotic-mediated engraftment (Frazão et al., 2022). Even in time scales of weeks with an estimated >6,000 bacterial generations, the authors uncovered both diversifying selection supporting coexistence of strains and directional selective sweeps which were determined to predominantly arise from new mutations and prophage acquisition, respectively. As bacterial strains are separated by spatial, temporal, and environmental elements, mutations, and gene loss events can reshape their genomes.

## Measuring and manipulating strain diversity

While many modern sequencing approaches are capable of resolving strains in theory, in practice, it is easier said than done. 16S rRNA gene sequencing typically lacks the ability to resolve strains and often species as well (Johnson et al., 2019). While full-length 16S rRNA sequencing may improve species resolution, it is still limited in its ability to meaningfully resolve strains due to the slow rate of evolution in the 16S rRNA gene versus the rest of the genome (Callahan et al., 2019). On the other hand, metagenomic sequencing offers the possibility to resolve strains with certain limitations. Owing to a desire for dimensional reduction and effective communication, metagenomic data are often summarized to higher taxonomic levels such as the species, genus, family, or even phylum. Instead, accessible methods are needed to meaningfully quantify strains, and perhaps more importantly, understand the biological significance of that strain variation.

Culture represents a traditional way through which strain diversity can be determined and quantified. Conventional culture methods are capable of isolating hundreds or thousands of strains with sufficient effort (Poyet et al., 2019; Hitch et al., 2021; Afrizal et al., 2022). Resulting colonies can then be dereplicated on the basis of MALDI-TOF profiles and/or fingerprinting approaches such as RAPD or ERIC PCR (Versalovic et al., 1991; Bazzicalupo and Fani, 1996). The resulting genomes of these strains can then be sequenced which will lead to assemblies almost invariably higher quality than those derived from metagenomic methods. This approach also allows for direct phenotyping and laboratory experimentation on strains. The advantages of this method are offset by the significant resources and infrastructure required to conduct these approaches at large scales and culture bias against many of the most prevalent members of the gut microbiome: the inability to effectively culture as many as 80% of the microbes found in the gastrointestinal tract (Lagier et al., 2012).

**Figure 2:**
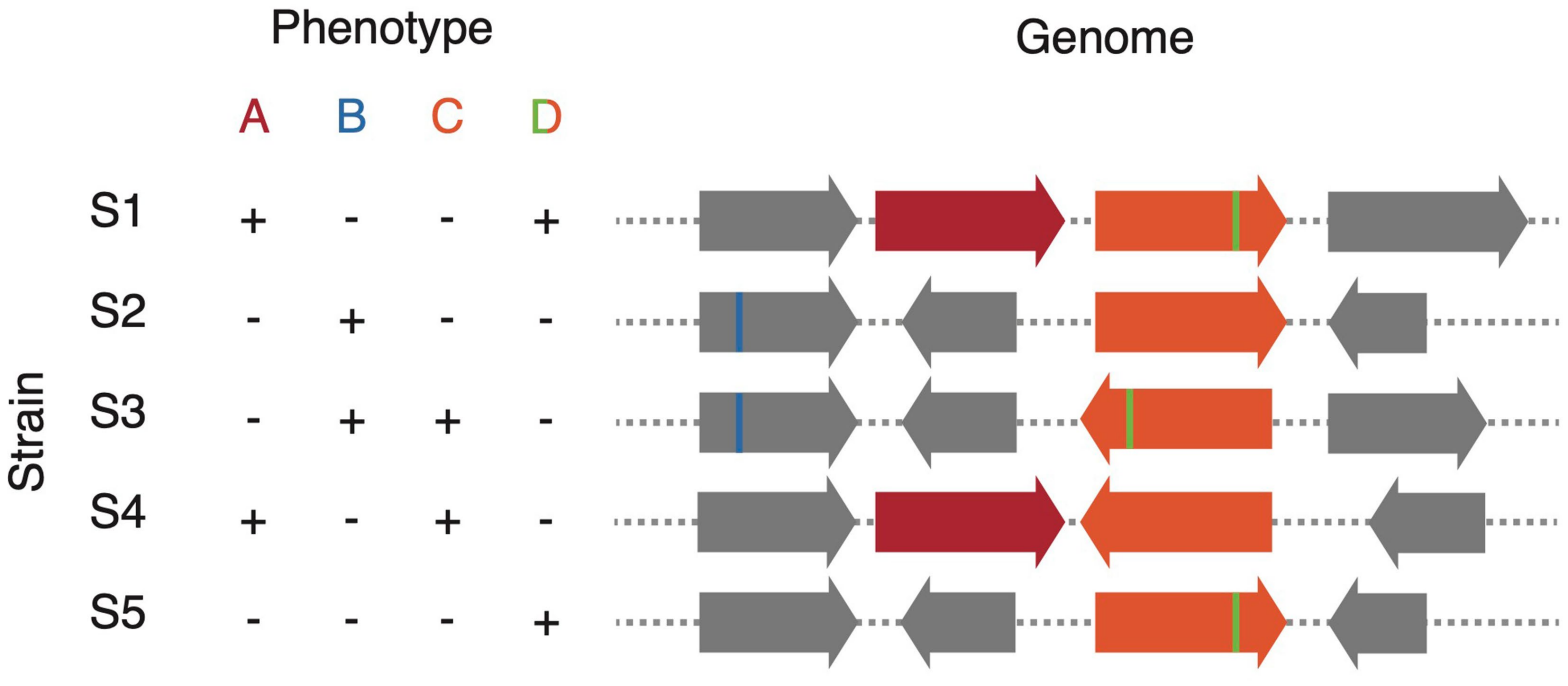
Schematic representation of comparative genomics analysis to match genotype to phenotype. Phenotype A results from the variable presence of a gene (red), phenotype B from a SNP (blue), phenotype C from a structural variant (orange), and phenotype D from a combination of variants: the simultaneous presence of structural variation (orange) and a SNP (green).

Where strain isolates are available in culture, strain variation can be a powerful tool for comparative genomic approaches to discover genes and enzymes of interest. Traditional screening methods, such as a transposon mutagenesis screen, require a genetically tractable host to randomly inactivate genes followed by screening of thousands of clones to look for phenotypic changes (Barquist et al., 2013). Alternatively, strain variation gives rise to what could be thought of as a “natural combinatorial knockout system.” In effect, if the trait of interest is variable among members of the species, which is often the case for traits involving host–microbe interactions, screening as few as 10 strains of the same species may be sufficient to map the genetic determinant (Bisanz et al., 2020; Alexander et al., 2022). A variety of comparative genomics approaches may be used for these analyses; however, it should be noted that the genetic determinants may be driven by any combination of: gene presence/absence, single-nucleotide polymorphisms (SNPs), and structural rearrangements (Figure 2). Gene presence/absence can be inferred relatively easily through methodologies employing reciprocal blast (Li et al., 2003; Lechner et al., 2011; Page et al., 2015); however, most methodologies to call SNPs are tailored to call SNPs in the core genome rather than those in the accessory. Alternatively, we have shown that the use of tiled *k*-mers is capable of accurately detecting explanatory SNPs and other structural changes; however, their use comes at a significantly greater computational overhead (Maini Rekdal et al., 2019; Bisanz et al., 2020). Because of the potential for combinations of predictors driving phenotype: for example, gene A or gene B, or the presence of a gene A plus the absence of a negative regulator gene C, machine learning approaches provide a powerful tool. Random Forest classifier/regression models are particularly adept at this task as they have straight forward metrics for each feature’s importance/predictive value and they generally consider combinations of explanatory variables in their decision trees (Chen and Ishwaran, 2012). Indeed, we have used variants of this approach across multiple manuscripts (Koppel et al., 2018; Maini Rekdal et al., 2019, 2020; Bess et al., 2020; Bisanz et al., 2020; Pröbstel et al., 2020; Alexander et al., 2022; Kyaw et al., 2022; Noecker et al., 2022; Paik et al., 2022). These comparative genomics approaches are attractive because phenotypes can be screened at a relatively low-throughput scale and they bypass the need for genetic tools. This immediately opens up new possibilities for the vast majority of gut microbes in which genetic manipulation is not yet possible.

Metagenomics is the current state of the art approach for cataloging strain variation at massive scales and for mapping strain dynamics within samples (Qin et al., 2010; Schloissnig et al., 2013). MAGs have allowed for unprecedented analysis of genetic diversity among gut microbiomes through binning genome fragments of an individual strain from the mixed population. This is typically accomplished through examining co-occurrence, co-abundance, and sequence composition of the fragmented assembly (Wang et al., 2015; Zhernakova et al., 2016). The use of MAGs does however have some important limitations as it often struggles to bin low coverage/low abundance organisms and there is a finite probability that when two strains are present within a sample at similar abundances, they will be combined into a single MAG (Chen et al., 2020). Current approaches can detect heterogeneity within genomes on the basis of variants and duplication of single copy genes; however, careful analysis and polishing is required (Parks et al., 2015; Mineeva et al., 2020), while new approaches to separate MAGs into separate strains are being developed (Quince et al., 2021). One challenge for the generation of high-quality MAGs from metagenomic data is limitations in sequencing technologies. Short-read sequencers are typically preferred due to lower error rates than long-read sequencers, but shorter reads are typically unable to resolve repetitive regions of genomes. This has led to the proliferation of hybrid assembly approaches combining both long-read and short-read approaches to improve metagenomic-binning and genome assembly (Bertrand et al., 2019); however, recent advances in the accuracy of long-read technologies are beginning to enable the generation of high-quality MAGs based on long reads alone (Sereika et al., 2022).

As with the comparative genomics approaches previously identified, SNPs provide a powerful tool for strain genotyping in metagenomic data (Truong et al., 2017; Shi et al., 2022). One method, StrainPhlAn, reconstructs SNPs within species-specific marker genes and uses these to infer strain-level phylogenies (Truong et al., 2017). A more recent approach, GenoTyper for Prokaryotes (GT-Pro) uses a less computationally intensive alignment-free method based on unique *k*-mers that are compared to a catalog of SNPs to more efficiently genotype strains (Shi et al., 2022).

Rapid advances in single-cell genomics technologies have brought exciting opportunities for microbiome research through physical and chemical methods to resolve single strain genomes. Flow-Assisted Cell Sorting (FACs) has been used to isolate single cells for use in single-cell sequencing (Rinke et al., 2014). More commonly, droplet-based microfluidics have been applied (Lan et al., 2017). The droplets trap individual bacterial cells from which sequencing libraries are then prepared at the single-cell scale (Hosokawa et al., 2017). Unfortunately, these methods often feature lower per-cell sequencing depth and require significant amplification to generate sufficient material for sequencing which limits the ability of these methods to resolve full genome assemblies. To aid in overcoming these limitations, single-cell sequencing and conventional metagenomic approaches may be combined to improve the quality and strain-level resolution (Arikawa et al., 2021). Alternatively chemical methods may be employed such as high-throughput chromosome conformation capture (Hi-C; Burton et al., 2014; Du and Sun, 2022). Originally developed for analyzing chromatin structure, DNA in close proximity, i.e., belonging to the same bacterial chromosome, is ligated together before sequencing, revealing which fragments are most likely to be from the same microbial genome (Pal et al., 2019).

## Strain diversity as determinant of microbiome assembly and function

The biological impact of strain variation is widely recognized in the field of bacterial pathogenesis wherein virulence traits are known to vary significantly within species. *Escherichia coli* represents perhaps the best-known example: members of this species can be the cause of severe diarrhea and dysentery (Kopecko et al., 1985), the most common cause of urinary tract infection (Johnson, 1991), or beneficial microbes administered intentionally as a probiotic to prevent diarrheal illness (Henker et al., 2007). Indeed, the virulence properties of many well-known bacterial pathogens vary significantly as a function of gene content and lineage including *Clostridioides difficile* (Hunt and Ballard, 2013), the *Bacillus cereus* group (Ceuppens et al., 2013), *Cutibacterium [Propionibacterium] acnes* (Tomida et al., 2013), and *Bacteroides fragilis* (Pierce and Bernstein, 2016). *Bacteroides fragilis* exists as one of the most common commensals of the human gut microbiome whose colonization is established early in life and may be vertically acquired; however, conventional wisdom would suggest that this species is an opportunistic pathogen (Carrow et al., 2020). *Bacteroides fragilis* strains exhibit the variable presence of a metalloprotease toxin which disrupts barrier function and drives intestinal inflammation (Moncrief et al., 1995); however, opposing this function, some strains produce an extracellular polysaccharide (polysaccharide A) that helps promote barrier function through modulation of regulatory T cells (Round and Mazmanian, 2010).

Strain variation also drives community composition through competitive exclusion *via* a variety of mechanisms. Ecological theory dictates that strains or species that occupy the same niche will compete resulting in exclusion from the community (Hardin, 1960). This has been observed in *Eggerthella lenta*, *Bacteroides* spp., and most recently *C. acnes* (Hecht et al., 2016; Bisanz et al., 2020; Conwill et al., 2022). While the skin can be colonized by multiple strains of *C. acnes,* individual pores contain a clonal strain population indicating that there may be a spatial component to strain variation across host-associated microbiomes (Conwill et al., 2022). These observations have implications for the gut microbiome in terms of diversity and the use of fecal microbiota transplants (FMTs) to treat diseases. One important aspect of competitive exclusion is the timing of a strain being introduced into the community as established strains and species are not as likely to be excluded from a community as newly introduced strains (Grainger et al., 2019; Munoz et al., 2022). This is particularly relevant as the gut microbiome is usually inherited from an individual’s mother (Mueller et al., 2015; Asnicar et al., 2017; Duranti et al., 2017). This has been experimentally demonstrated with *Akkermansia muciniphila* strains, wherein mice colonized with one strain were resistant to colonization by a second (Munoz et al., 2022). However, strict strain homogeneity through competitive exclusion is not always the case, and some strains can co-exist as experimentally demonstrated for *Phocaeicola [Bacteroides] vulgatus* and *E. lenta* (Bisanz et al., 2020; Munoz et al., 2022). Acquisition of genes allowing for exploitation of a new nutrient source may partially alleviate this competition as has been engineered into *Bacteroides* spp. (Shepherd et al., 2018).

The ability of microbes to illicit immune responses can also be highly strain specific. Various strains of *Ruminococcus gnavus, A. muciniphila,* and *B. fragilis* can have wide reaching effects on modulation of the immune system (Troy and Kasper, 2010; Cassard et al., 2016; Henke et al., 2021; Liu et al., 2021). *Ruminococcus gnavus* shows strain-level differences in immune response with distinct immune responses found depending on the presence of a biosynthetic pathway for production of a capsular polysaccharide (Henke et al., 2021). Similarly, *A. muciniphila* displays variable anti-inflammatory effects through unknown mechanisms (Zhai et al., 2019; Liu et al., 2021). A range of immune responses are also detected with *Lactobacillus paracasei* strains which can have a range of inhibiting activation of mouse mast cells and human basophils (Cassard et al., 2016).

Other than virulence, perhaps, the best-known examples of strain diversity are in drug-microbe interactions. It is becoming increasingly acknowledged that there are extensive interactions between gut microbes and orally consumed drugs/xenobiotics (Maier et al., 2018; Zimmermann et al., 2019; Klünemann et al., 2021). These drug metabolism traits are particularly interesting as we are quickly gaining answers as to how microbes can metabolize drugs, but not why. In most cases, we have not determined a fitness advantage from drug metabolism which creates the perfect opportunity for these pathways to largely exist in the accessory genome. Indeed, we were inspired by early work examining strain variation in *Lactobacillus* spp. (Douillard et al., 2013; Smokvina et al., 2013) to map variation in *E. lenta*, a highly prevalent, but relatively understudied member of the gut microbiome (Koppel et al., 2018). *Eggerthella lenta* was known to metabolize the cardiac drug digoxin as early as the 1980s (Dobkin et al., 1982); however, the mechanisms were unknown until 2013 when RNA-seq revealed an operon whose expression was induced by the presence of the drug (Haiser et al., 2013). Using comparative genomics approaches, we identified that this operon was part of a gene cluster which was variably present across strains of the species which explained why the presence of *E. lenta* was insufficient to predict digoxin metabolic activity (Koppel et al., 2018). Taking this a step further, we later determined that a single coding variant of the active enzyme CGR2 dictated its activity resulting in three phenotypes in effect: no metabolism, low metabolism, and high metabolism. Similarly, *E. lenta* operates in a meta-organismal pathway leading to the production of phytoestrogens which we mapped to the presence of a single enzyme variably present in the genome (Bess et al., 2020). *Eggerthella lenta* also cooperates with *Enterococcus faecalis* in the premature breakdown of the Parkinson’s drug levodopa which is determined by a SNP affecting enzyme activity (Maini Rekdal et al., 2019). Understanding the mechanisms of strain-level variation in drug interactions opens up new possibilities for precision medicine and pharmacological therapy: i.e., rather than try to sequence microbiome composition or quantify specific microbes, we could instead design targeted assays to predict drug metabolism based on detection/quantification of specific genes or variants.

## Concluding remarks

Advancing microbiome science from a descriptive to a mechanistic science requires a detailed understanding of microbial function, but these functions are often not conserved at the species level. If we stereotype all *E. coli* as pathogens, or *A. muciniphila* as beneficial, we are likely to miss the trees for the forest. By viewing our data through a taxonomic lens, we may lose the ability to find the important determinants of microbiome structure and function. We need to be aware of strain variation in our data and carefully catalog it. Recent advances in sequencing technology are making it quickly possible to follow strains in metagenomic samples, but we then need databases incorporating functional annotations and phenotypic information to draw mechanistic insight from this data. By pairing these approaches with wet lab experimentation, we can turn strain variation from one of the major challenges in microbiome research to one of its greatest tools.

## Author contributions

All authors listed have made a substantial, direct, and intellectual contribution to the work and approved it for publication.

## Conflict of interest

The authors declare that the research was conducted in the absence of any commercial or financial relationships that could be construed as a potential conflict of interest.

## Publisher’s note

All claims expressed in this article are solely those of the authors and do not necessarily represent those of their affiliated organizations, or those of the publisher, the editors and the reviewers. Any product that may be evaluated in this article, or claim that may be made by its manufacturer, is not guaranteed or endorsed by the publisher.
